# Supplementary material for: Functional unknomics: Systematic screening of conserved genes of unknown function
Source: PLoS Biol. 2023 Aug 8;21(8):e3002222. doi: 10.1371/journal.pbio.3002222 (PMC10409296; doi:10.1371/journal.pbio.3002222)
Supplement: S1 Text — (PDF) [file pbio.3002222.s005.pdf]

Supplemental Text - Statistical methods

Here we provide further details to complement the outline of the statistical methods presented in the Methods section. The approach used consists of three steps: modelling the data to associate each gene with statistical parameters; construction of an outlier region in the space of these parameters; and performing hypothesis tests to determine whether these parameters fall within this region. The material here is similarly organised into three sections describing each of these steps. In much of what follows, we consider the experimental setting (i.e. fertility, wing size etc.) to be fixed and describe general procedures that we apply, with some modifications, to each of the settings.

1 Modelling the data

1.1 General procedure

The data for each experiment takes the general form $(Y_{ij}, x_{ij}) \in \mathbb{R}^d \times \mathbb{R}^p$, $i = 1, \dots, n_j$, $j = 1, \dots, J$, where J was the total number of genes, and $d \in \{1,2\}$. Here Y_{ij} corresponds to the ith measurement taken on the jth gene and the x_{ij} are associated covariates that may indicate the batch in which the measurement was taken, for example. Our goal is to identify outlying genes, and for this purpose we first construct a parametric model for the data of the form

$$Y \sim F(\theta, \eta, X)$$

where Y and X collect together the response of covariates respectively, $\theta = (\theta_1, \dots, \theta_J) \in \mathbb{R}^{d \times J}$ are the parameters associated the with genes and η represents a collection of nuisance parameters (e.g. parameters associated with the different batches). The statistical problem at hand then is to identify outlying θ_j. For this we need to introduce a notion of what it means to be an outlier, and then propose a methodology for testing for each j whether θ_j is an outlier. These latter two tasks are described in Sections 2 and 3. To do these, we require estimates $(\hat{\theta}_j)_{j=1}^J$ of $(\theta_j)_{j=1}^J$ that are approximately unbiased and Gaussian with estimated variance $\hat{\Sigma} \in \mathbb{R}^{(d \cdot J) \times (d \cdot J)}$. Note that as we are only interested in differences between different θ_j, we are for example free to introduce a sum-to-zero constraint on these parameters to reduce the overall variance, and we do this throughout.

Below we present the specific statistical models F used for each of the different experimental datasets for which (versions of) maximum likelihood estimation then delivers these quantities. All computations were performed in R [1].

1.2 Fertility

Let $Y_{ijk} \in \mathbb{Z}$ be the ith brood size measurement corresponding to female flies with gene type j in batch k for $i = 1, \dots, n_{jk}$ (where n_{jk} may be 0 for some (j,k)). We will first present our analysis of the data on females; analysis for the data on males proceeded similarly.

To examine the mean–variance relationship (i.e. how $\mathbb{E}(Y_{ijk})$ relates to $\mathrm{Var}(Y_{ijk})$), we first formed for all (j,k) such that $n_{jk} \geq 2$,

$$m_{jk} = \frac{1}{n_{jk}} \sum_{i=1}^{n} Y_{ijk}, \qquad s_{jk} = \frac{1}{n_{jk}} \sum_{i=1}^{n} (Y_{ijk} - m_{jk})^2.$$

We then regressed m_{jk} on to s_{jk} via the following optimisation:

$$\hat{\beta} = \underset{\beta \in \mathbb{R}^2}{\arg\min} \sum_{(jk): n_{jk} \geq 2} n_{jk} \, |s_{jk} - \beta_1 m_{jk} - \beta_2 m_{jk}^2|.$$

The lack of intercept in this regression encodes the restriction that when $\mathbb{E}(Y_{ijk}) = 0$ we must have $\mathrm{Var}(Y_{ijk}) = 0$; the use of the absolute value rather than the more usual squared error loss is to account for the exponential-type tails we may expect for the s_{jk}; and the weights n_{jk} reflect the variance of the s_{jk}.

We thus obtained an estimated variance function $\hat{V}(\mu) = \hat{\beta}_1 \mu + \hat{\beta}_2 \mu^2$ such that $\hat{V}(\mathbb{E}Y_{ijk}) \approx \mathrm{Var}(Y_{ijk})$. We obtained coefficients

$$\hat{\beta}_1 = 8.229653 \qquad \hat{\beta}_2 = -0.04984021,$$

and as $\hat{\beta}_2$ was negative, we were able to express $\hat{V}$ as a scaled version of a Bernoulli variance

function via

$$\hat{V}(\mu) = \tilde{V}(\tilde{\mu}) = \frac{\hat{\beta}_1^2}{|\hat{\beta}_2|}\tilde{\mu}(1-\tilde{\mu})$$

with $\tilde{\mu} = |\hat{\beta}_2|\mu/\hat{\beta}_1$. To fit a regression model with this form of variance function, we used a quasi-binomial regression after transforming the data $Y_{ijk} \mapsto \tilde{Y}_{ijk} = |\hat{\beta}_2|Y_{ijk}/\hat{\beta}_1$. The transformed data took values in $[0,1]$ so we used a logit link and modelled the mean $\mathbb{E}\tilde{Y}_{ijk}$ as

$$\log\left(\frac{\mathbb{E}\tilde{Y}_{ijk}}{1-\mathbb{E}\tilde{Y}_{ijk}}\right) = \text{logit}(\mathbb{E}\tilde{Y}_{ijk}) = \theta_{j1} + \eta_{k1}.$$

To handle zero counts we used the bias correction of [2], as implemented in [3] , which always produces finite parameter estimates. The analysis of the male data was very similar, and in the end we obtained estimates $(\hat{\theta}_j)_{j=1}^J$ and block diagonal estimated variance matrix $\hat{\Sigma}$ (as the male and female data were independent).

1.3 Wing size

Let $Y_{ijk} \in \mathbb{R}^2$ be the ith measurement on the jth gene in the kth batch, defined for $i = 1, \ldots, n_{jk}$ (where n_{jk} may be 0 for some (j,k)) with first and second components denoting measurements for anterior and posterior wing segments respectively. We used the model

$$Y_{ijk} = \theta_j + \eta_k + \varepsilon_{ijk}$$

where $\varepsilon_{ijk} \overset{i.i.d.}{\sim} \mathcal{N}(0, \Sigma_j)$ with $\Sigma_j \in \mathbb{R}^{2\times 2}$. Inspection of the data showed that the correlation matrices corresponding to the Σ_j vary very little over j and the difference is barely detectable by permutation tests. We therefore constrained Σ_j in the following way: $\Sigma_j = D_j^{1/2}\Sigma_{\text{univ}}D_j^{1/2}$ where $\Sigma_{\text{univ}} \in \mathbb{R}^{2\times 2}$ is a universal correlation matrix and the $D_j \in \mathbb{R}^{2\times 2}$ are diagonal matrices with variances corresponding to each gene.

1.4 PolyQ aggregates

We first constructed from the available data two quantities from each replicate: the number of aggregates with area in pixels greater than 50, and the corresponding number with area less than or equal to 50. They form the components of $Y_{ijk} \in \mathbb{Z}^2$, for which we use quasi-Poisson models with $\log$ links as follows.

$$\begin{pmatrix}\log(\mathbb{E}Y_{ijk1})\\ \log(\mathbb{E}Y_{ijk2})\end{pmatrix} = \theta_j + \eta_k.$$

We performed two separate Poisson regressions for each component of the response. In order to avoid issues where parameter estimates from standard maximum likelihood estimation were too large, we employed the bias correction of [2], as implemented in [3]. To estimate the covariance matrix of the parameters, we noted that the working residuals from the regressions displayed a covariance that was constant across fitted values from each of the regressions. Using this estimated covariance and estimated dispersion parameters we formed a full covariance matrix $\hat{\Sigma} \in \mathbb{R}^{(2\cdot J)\times(2\cdot J)}$ for all $(\hat{\theta}_j)_{j=1}^J$.

1.5 Survival under stress

Let Y_{ijkl} and T_{ijkl}^Y denote the censored survival and censoring times under oxidative stress for the ith replicate of gene j in batch k and wheel l. We fitted a Cox proportional hazards model of the form

$$h_{ijkl}(y) = \exp(\theta_j + \eta_l)h_k(y),$$

where h_{ijkl} is the hazard function of the unobserved uncensored version Y_{ijkl}^* of Y_{ijkl}, and h_k is an unspecified baseline hazard function for batch k.

We used an analogous model for the data concerning survival times under starvation.

1.6 Climbing speed

Let Y_{ijkl} and Z_{ijkl} denote the ith speed measurement corresponding to gene j, batch k and repeat l

for days 8 and 22 respectively. We used the following random effects models:

$$Y_{ijkl} = \theta_{j1} + \eta_{k1} + \zeta_{jkl1} + \varepsilon_{ijkl1}$$

$$Z_{ijkl} = \theta_{j2} + \eta_{k2} + \zeta_{jkl2} + \varepsilon_{ijkl2}$$

where $\zeta_{jklm} \sim \mathcal{N}(0, \sigma_{km}^2)$ and $\varepsilon_{ijklm} \sim \mathcal{N}(0, \sigma_m^2)$, all independently.

2 Outlier region construction

From the initial regression, we obtained estimates $(\hat{\theta}_j)_{j=1}^J$ for the parameters $(\theta_j)_{j=1}^J$ corresponding to each gene, and their associated estimated variance matrix $\hat{\Sigma} \in \mathbb{R}^{(d \cdot J)\times(d \cdot J)}$. In order that an elliptical outlier region was appropriate, we transformed the estimates depending on their distribution to give $\hat{\mu}_j = f(\hat{\theta}_j)$ where the transform function $f: \mathbb{R}^d \to \mathbb{R}^d$ chosen is given in Table 1.

**Dataset**	**Transform function f**
Fertility	logistic
Wing size	identity
PolyQ aggregates	exponential
Survival under Stress	identity
Climbing speed	identity

Table 1; Transform functions used for different datasets.

Let us write $\mu_j = f(\theta_j)$ for each $j = 1, \dots, J$. We considered f as fixed, and as is common in the analysis of outliers, considered a model for the μ_j as samples from a mixture of a normal distribution and an outlier distribution F_{out} [4]:

$$\mu_j \sim \gamma\mathcal{N}(\mu, \Sigma_\mu) + (1-\gamma)F_{\text{out}}.$$

We assumed the mixture proportion γ to be greater than 0.5 and that the support of F_{out} was sufficiently far from μ. We used the minimum covariance determinant estimator [5], as implemented in [6], to give a robust estimate $\hat{\mu}$ of μ and an initial estimate $\check{\Sigma}_\mu$ of Σ_μ. Whilst we can expect that $\hat{\mu}$ is a reasonable estimate of μ, $\check{\Sigma}_\mu$ will be substantially inflated by the sampling variability of the $(\hat{\mu}_j)_{j=1}^J$. To correct for this, we employed the following bootstrap strategy.

1. Produce bootstrap samples $(\hat{\theta}_j^{(b)})_{j=1}^J \sim \mathcal{N}((\hat{\theta}_j)_{j=1}^J, \hat{\Sigma})$ for $b = 1, \dots, B$.
2. Form $\hat{\mu}_j^{(b)} = f(\hat{\theta}_j^{(b)})$ for $j = 1, \dots, J$ and $b = 1, \dots, B$.
3. Compute robust covariance estimates $\check{\Sigma}_\mu^{(1)}, \dots, \check{\Sigma}_\mu^{(B)}$ based on each of the bootstrap samples $(\hat{\mu}_j^{(1)})_{j=1}^J, \dots, (\hat{\mu}_j^{(B)})_{j=1}^J$ using the minimum covariance determinant estimator.
4. Set

$$\bar{\Sigma}_\mu = \frac{1}{B}\sum_{b=1}^{B} \check{\Sigma}_\mu^{(b)}$$

and finally define our final estimate $\hat{\Sigma}_\mu$ of Σ_μ by

$$\hat{\Sigma}_\mu = \check{\Sigma}_\mu^{1/2}\bar{\Sigma}_\mu^{-1/2}\check{\Sigma}_\mu\bar{\Sigma}_\mu^{-1/2}\check{\Sigma}_\mu^{1/2}.$$

The rationale for this approach is that

$$H: \mathbb{R}^{d\times d} \to \mathbb{R}^{d\times d}$$

$$\Omega \mapsto \check{\Sigma}_\mu^{1/2}\bar{\Sigma}_\mu^{-1/2}\Omega\bar{\Sigma}_\mu^{-1/2}\check{\Sigma}_\mu^{1/2}$$

is a mapping that satisfies $\hat{\Sigma}_\mu = H(\check{\Sigma}_\mu)$ and

$$\frac{1}{B}\sum_{b=1}^{B} H\left(\check{\Sigma}^{(b)}\right) = \check{\Sigma}_\mu.$$

Thus, we can think of H as a corrective transformation that were $(\hat{\mu}_j)_{j=1}^J$ to be a sample from the ground truth, gives an approximately unbiased estimate of its (robust) covariance. Applying H to $\check{\Sigma}_\mu$ should similarly correct it to give a better estimate of Σ_μ. The reason for generating the bootstrap samples at the level of the untransformed parameters is that the Gaussian approximation in step 1

of the procedure above, which mimics the sampling distribution of the $(\hat{\theta}_j)_{j=1}^J$, would typically be more reliable than the analogous approximation for the $(\hat{\mu}_j)_{j=1}^J$.

Given our final estimates $\hat{\mu}$ and $\hat{\Sigma}_\mu$, we set the outlier region to be the complement of the elliptical contour of a $\mathcal{N}_d(\hat{\mu}, \hat{\Sigma}_\mu)$ density such that the probability of $\zeta \sim \mathcal{N}_d(\hat{\mu}, \hat{\Sigma}_\mu)$ falling within the region is given by 0.05 or 0.1, depending on the dataset. This outlier region can be mapped to the θ-space using the inverse of f; in the sequel we will refer to this region as R.

3 Testing for outliers

Given outlier region R such that all j for which $\theta_j \in R$ are deemed outliers, we constructed for each j an (approximate) p-value p_j for the null hypothesis $\theta_j \notin R$. In the cases where the region was an interval, this was straightforward. In the cases where the region was two-dimensional, this was done using a bootstrap scheme, the main steps of which were as follows. Denote by A the complement of R, and also let A_μ be the (elliptical) region $f(A)$.

1. Compute via the delta method an estimate $\hat{\Omega}_j$ of the variance of $\hat{\mu}_j$.
2. Compute the projection $\tilde{\mu}_j$ of $\hat{\mu}_j$ on to the elliptical region A_μ using the Mahanolobis distance with covariance $\hat{\Omega}_j$:
$$\tilde{\mu}_j = \operatorname*{arg\,min}_{m \in A_\mu} (\hat{\mu}_j - m)^T \hat{\Omega}_j^{-1} (\hat{\mu}_j - m).$$
(Details for how this is performed are given in Section 4.)
3. Set
$$T_j = (\hat{\mu}_j - \tilde{\mu}_j)^T \hat{\Omega}_j^{-1} (\hat{\mu}_j - \tilde{\mu}_j).$$
Also define $\tilde{\theta}_j = f^{-1}(\tilde{\mu}_j)$.
4. Let $\tilde{\Sigma}_j$ be an estimate of the maximum likelihood estimate of θ_j under the null that $\theta_j \in A$. Generate $B = 100000$ bootstrap samples $\tilde{\theta}_j^{(1)}, \ldots, \tilde{\theta}_j^{(B)} \overset{i.i.d.}{\sim} \mathcal{N}_d(\tilde{\theta}_j, \tilde{\Sigma}_j)$. Let $\tilde{\mu}_j^{(b)} = f(\tilde{\theta}_j^{(b)})$.
5. Compute bootstrap versions of the test statistic T_j:
$$T_j^{(b)} = \min_{m \in A_\mu} (\tilde{\mu}_j^{(b)} - m)^T \hat{\Omega}_j^{-1} (\tilde{\mu}_j^{(b)} - m).$$
6. Then
$$p_j = \frac{\sum_{b=1}^B \mathbb{1}_{\{T_j^{(b)} \geq T_j\}}}{B}$$
in a Monte Carlo estimate of the p-value. To improve the quality of this estimate, we in fact used an importance sampling scheme where initially the $\hat{\theta}_j^{(b)}$ were generated from a mixture of the Gaussian distribution above, and $\mathcal{N}_d(\hat{\theta}_j, \tilde{\Sigma}_j)$ (with mixture proportions 0.5); the $\mathbb{1}_{\{T_j^{(b)} \geq T_j\}}$ terms were then weighted according to the importance sampling weights..

The rationale for this is as follows. The test statistic T_j encapsulates how far $\hat{\mu}_j$ is from the region A_μ taking into account the variance of the $\hat{\mu}_j$ (directions in which $\hat{\mu}_j$ is highly variable are effectively down-weighted). Under the null hypothesis that $\theta_j \in A$, we should have $\tilde{\mu}_j \approx \mu_j : f(\theta_j)$ and so the bootstrap distribution should approximate the null distribution and thus provide effective calibration for T_j.

We finally apply false discovery rate (FDR) correction to the p-values using the Benjamini–Hochberg procedure [7]. Although controlling for batches and the fact that the outlier region is determined using the data would make the p-values dependent, the dependence should be weak and thus the Benjamini–Hochberg procedure should at least approximately control the FDR.

4 Ellipse projection

Here we describe an efficient approach to computing
$$x^* = \operatorname*{arg\,min}_{x \in A} (x - z)^T M (x - z)$$

where $M \in \mathbb{R}^{d \times d}$ is a symmetric positive definite matrix and ellipsoid $A = \{x: x^T \Omega x \leq c\}$ for symmetric positive definite $\Omega \in \mathbb{R}^{d \times d}$, $c > 0$ and $z \notin A$. Equivalently, the problem is to find the minimum $c^* > 0$ such that there exists x^* with

$$(x^* - z)^T M (x^* - z) \leq c^* \text{ and } (x^*)^T \Omega x^* \leq c.$$

By Lagrangian duality, we know there exists λ that

$$x^* = \arg\min_{x \in \mathbb{R}^d} \{(x - z)^T M (x - z) + \lambda x^T \Omega x\}.$$

Consider the eigendecomposition $M = PD^2P^T$. Writing $y^* = DP^T x^*$ we have

$$y^* = \arg\min_{y \in \mathbb{R}^d} \{\| y - DP^T z \|_2^2 + \lambda y^T D^{-1} P^T \Omega P D^{-1} y\}.$$

Let the eigendecomposition of $D^{-1}P^T \Omega P D^{-1}$ be $U \Lambda U^T$. We see that then

$$z^* = (I + \lambda \Lambda)^{-1} U^T D P^T z$$

where $z^* = U^T y^*$ so $x^* = PD^{-1}Uz^*$ and λ is such that $(z^*)^T \Lambda z^* = c$.

References

1. R Core Team. R: A Language and Environment for Statistical Computing. In: https://www.r-project.org/. 1 Jan 2018.

2. Firth D. Bias reduction of maximum likelihood estimates. Biometrika. 1993;80: 27–38.

3. Kosmidis I. brglm: Bias reduction in binary-response generalized linear models. In: https://cran.r-project.org/web/packages/brglm/index.html. 1 Jan 2019.

4. Hawkins DM. Identification of Outliers. Springer; 1980. p. 194. Available: https://link.springer.com/book/10.1007/978-94-015-3994-4

5. Rousseeuw PJ, Driessen KV. A fast algorithm for the minimum covariance determinant estimator. Technometrics. 1999;41: 212–223. doi:10.1080/00401706.1999.10485670

6. Maechler M, Rousseeuw PJ, Croux C, Todorov V, Ruckstuhl A, Saliban-Barrera M, et al. robustbase: Basic robust statistics. In: http://robustbase.r-forge.r-project.org. 1 Jan 2018.

7. Benjamini Y, Hochberg Y. Controlling the false discovery rate: a practical and powerful approach to multiple testing. J Royal Stat Soc B. 1995;57: 289–300.
