# Supplementary material for: Functional unknomics: Systematic screening of conserved genes of unknown function
Source: PLoS Biol. 2023 Aug 8;21(8):e3002222. doi: 10.1371/journal.pbio.3002222 (PMC10409296; doi:10.1371/journal.pbio.3002222)
Supplement: Attachment — Submitted filename: Response to Reviewers D-23-00105R1.pdf [file pbio.3002222.s012.pdf]

Response to Reviewers' Comments.

We are very grateful indeed to the reviewers for their positive comments about our work and their constructive suggestions for improvements. We have followed their suggestions and added analysis and discussion as described below.

Reviewer #1:

The manuscript by Rocha et al. provides two thrusts to facilitate studies of little investigated genes. In the first thrust they rank genes according to the extent of prior annotation. In the second thrust they provide functional insight into several little studied genes.

The main novelties of the first thrust lie in the inclusion of annotations across diverse organisms, and in creating a tool (interactive database) that would allow other researchers to weight the impact of distinct annotations toward knownness according to their preference. The main novelties of the second thrust lie in the scope of the considered understudied genes and the diversity of phenotypic assays. Aside from the two thrusts, the manuscript consolidates the insight that several unstudied genes have some physiological relevance, and coins the phrase "unknomics" for a yet unnamed approached to targeted gene scholarship.

On the technical side, would like to note that GO can undergo changes across different years that remove annotations (Gillis et Pavlidis, 2013). Therefore, I suggest some extra words on whether temporal trends that are shown in the manuscript are based on the GO release of the indicated years, or based on a retrospective inspection of a current release. This might also affect the conclusion on knownness necessarily decreasing over years.

We apologise for not making it clear that the temporal trends are based on the retrospective inspection of a current release. This seemed most informative as annotations that have been removed are not likely to have contributed to knownness. A sentence explaining this has been added to the Materials and Methods section: "*Note that this information only covers current entries and so annotations made in the past that were subsequently removed are not included in analyses of the change in knownness.*"

Further, GO annotations can themselves be informed by knowledge about orthologs. Thus, knownness score may be a composite of annotations and organisms with orthologs. To better frame conclusions, particularly on genes with low knownnness only being present in some other model organisms (Fig 2D), I would have found helpful a supplemental panel that compared knownness scores against the number of orthologs in other species.

This is an interesting point and we have now added a Supplemental panel that compares knownness with the number of species that contain an ortholog (Supplemental Figure 2D). As the reviewer suspects, the number of orthologs for each cluster tends to increase with the knownness scores, and this is now noted in the text.

Reproducibility could be increased by adding the release number of GO or download date to the methods section.

We agree that this should have been added, and it is now included in the Materials and Methods (GO Release 2022-09-19).

The main text and abstract could be more transparent in conveying the absolute number of hits of distinct assays. Similarly, the observed verification rate is around 50% (which is estimated from a small number of genes, and could thus in reality be even higher or lower given the statistical uncertainty around a small number of sampling events; also the verification rate well differ for some of the distinct assays used here). Thus, providing an uncorrected estimated of the share of genes with phenotypes (as for viability in the current abstract) could lead to an inflated perception of the magnitude of the findings. As it does not seem necessary for this manuscript that many hits have phenotypes, and as others have already reported on the importance of unstudied genes, some more

moderate presentation would in my view be preferable as some readers might not be experienced with Drosophila hairpin screens.

Based on my own contribution to a genome-wide tissue-specific in-vivo Drosophila hairpin screen and the design of secondary and tertiary screens, I would similarly personally shy away from findings that seem derived from a single screen and/or biological replicates (experimental week, batch of food etc.). I am not advocating for more experimental work here, as there already is a lot of work that went into the experiments presented here, and the initial leads presented here seem very reasonable and useful to encourage and direct a further exploration of these genes. As the potential readership that extends beyond Drosophila specialists, would encourage an extra sentence or two that help readers not familiar with Drosophila how to engage with the specific screen results presented here.

To address these points we have replaced the phrase "*About a quarter are required for viability..*" in the Abstract with *"Knockdown of some genes resulted in loss of viability…"*. In the main text where we first discuss the results of the RNAi screen we have added a cautionary sentence as follows: "*In considering the results from RNAi screens one must always be mindful of off-target effects, and in Drosophila the possible effects of variability in genetic background and conditions of rearing and maintenance."*

In terms of presentation, I greatly enjoyed the smart idea of marking the human gene symbol next to the Drosophila gene symbol as this will increase findability through search engines. However, it seems that for some genes this scheme has not been applied yet.

We apologise for this inconsistency and have now gone through the text to ensure that in every case when a *Drosophila* gene symbol is provided, the human gene is also stated, either in brackets immediately after the gene or elsewhere in the sentence discussing the gene.

Fig 1E seems to indicate a "rich-get-richer" effect. An alternative presentation of the underlying data, which would promise to emphasize the value of unknomics and thus the present manuscript would be to test and show (very expected based on own side observations) that genes with fewer annotations will gain additional annotations at a slower absolute rate than well annotated genes.

This is a good idea, and so we have added an additional panel that shows that the genes with fewer annotations gain additional annotations and hence knownness at a slower rate than those genes that are well known (Supplementary Figure 2C).

I would find it helpful to see a graphical summary of genes vs. screens. For instance, a clustermap could show whether little investigated genes are pleiotrophic, or have very specific phenotypes. Similarly it would be interesting to see which share of the screened genes have no detected phenotype. Such a meta-analysis could possibly also be extended to see if certain pre-existing annotations (no matter how few) are informative on the phenotypes. Acknowledging that this manuscript seems to be under consideration for a brief report, presenting some of the present findings as a meta-analysis might also allow to shorten some of the text and main figures devoted to the specific screens.

Although there is a summary of the genes found in each screen in Supplemental Table 2, we agree that a graphical summary would be an effective means of providing an overview of the results. Thus, we have included such a cluster map as a supplemental figure (Figure S4A).

Related to the above point of contextualizing findings, a comparison to GenomeRNAi, which aggregates Drosophila hairpin screens, or a comparison to the murine IMPC phenotype database, could help to convey the relative advance brought forward by targeted screens on under-investigated genes over the mining of existing data. To guide further studies of unstudied genes either outcome would be a useful insight.

This is an interesting point, and indeed there are quite a lot of different genome-wide resources which might provide clues about protein function, but each has its own strengths and weaknesses. For instance, GenomeRNAi has not been adding new screens for the last ~6 years and the search function does not appear to work any longer. The IMPC is undoubtedly a powerful resource, and one we mention in the paper, but it covers only 39%

of genes, and in mice there are the problems associated with partially redundant paralogues. There are also further resources that are potentially useful such as the DepMap project on gene knockouts in cancer cell lines, and indeed we are currently exploring the use of machine learning to combine information from various such resources to predict gene function. Given all this, and the fact that using genome-wide resources to predict gene function is a well-established approach, we feel that an in-depth discussion of these resources and their potential use is too large a topic to include in what is already quite a long paper.

Lastly, the manuscript appears to focus on genes with a low knownness score in Drosophila. This invites the possibility that those genes that have a phenotype in specific assays might already be well known in other organisms (including humans). While again either outcome would be useful for guiding future studies into unstudied genes, I would be thrilled to learn whether the value of functional unknomics lies in adding information to unstudied genes of model organisms, or whether findings on unstudied genes of model organism could (still) provide functional insight into unstudied human genes.

We apologise that our text did not make it clear that the *Drosophila* genes we focused on are from clusters of orthologs selected because little was known about them in humans or any of 11 model organisms. The knownness score of a cluster is defined as the knownness of the best understood gene in that cluster (see Figure 1C), and so a low knownness score means that little is known about the gene in other organisms including humans. We have checked the text to make sure that this is clear and added a title to Figure 1C. We are certainly convinced that *Drosophila* and other model organisms still have plenty of scope to provide insight into unstudied human genes, and have added a note to this effect to the Discussion: "*This is also a reminder that studies in model organisms such as Drosophila still have the scope to provide insight into unstudied human genes*."

Reviewer #2:

In this interesting manuscript, Rocha et al. pinpoint the importance of the unstudied genome (or "unknome") suggesting that poorly studied proteins are not necessarily less important than widely studied ones. To facilitate studies about unknown proteins, the authors have assembled a database of proteins from several organisms and assigned a knowledge value based on the number of previous studies on that specific protein. Then they have a tested in drosophila the function of 260 genes that were previously poorly studied and have identified phenotypes in several assays, ranging from fertility to starvation resistance. The authors should consider the following points:

-Please provide the raw data for all the screens that have been performed as supplementary material. Table S2 currently reports only a qualitative description of only the RNAi that scored in the different assays. This is insufficient: please provide numerical values for all the assays that have been done and are shown in Fig. 3-5 and for all the lines that have been screened, including those that did not score in the specific assay. Please also indicate how many times and with how many flies the assay was done. Please clearly specify what RNAi stock has been used. The stocks have been identified as "JS" stocks but this does not correspond to any official naming of the VDRC stocks or any other collection.

Table S2 shows just a summary of the hits from all of the screens, with the raw data for the screens being provided in the supplementary files that are named S1 and S2 Data following what appears to be standard practice in PLOS Biology papers for data used to generate plots. We had only referred to these Data files in the figure legends and methods section, and so for clarity we have now added a reference to them in the section of the Results that introduces the functional screens. For all genes in the screens, the official VDRC name for the RNAi stock is provided in S1 Table (column C, which links out to the VDRC database), along with the gene's CG number and FlyBase FBgn ID.

- Please provide information for the negative and positive controls that were used in each RNAi screen (currently there are some indicated only for some assays). I guess "Pink" used as control for the climbing speed is "Pink1"?

We have corrected Pink to Pink1 in Figure 5B. As for the other controls, we initially incorporated these to help set up some assays we had not used before, and these are shown for information. However, we did not incorporate such controls in all the assays as we did not rely on control stocks for statistical determination of outliers, but rather relied on the much larger amount of information provided by the variation amongst all of the lines that were screened as this covers many more lines and so gives a more accurate measure of the degree of variation that is intrinsic in the assay and hence allows a more reliable means to identify outliers. This is a commonly used approach in large-scale RNAi screens and is briefly outlined in the Results with the details of the statistical analysis summarised in the Materials and Methods and provided in full in the statistical supplement.

- Based on the screens performed, is there any correlation between the degree of conservation between drosophila and human and the probability of uncovering a phenotype?

We have compared the degree of conservation for the genes that gave a phenotype to those that did not and found that there is a large range in both sets with no clear difference in the mean values. This is now mentioned briefly in the text and the plot shown as Figure S4B.

- There is large-scale interactome data for drosophila, mice, human, and other organisms. The authors may want to consider integrating this into the unknome database. For this paper, is there any correlation between protein-protein interactions and uncovering a phenotype in the screens? For example, do the proteins that scored in the assays interact with previously-studied proteins involved in the same processes?

We agree that protein-protein interactions can provide valuable insight into function. However, there are also a lot of false positives in high-throughput screens, and interactions that have been validated and published are very likely to have been incorporated into GO and thus already added to knownness. In addition, there are other sources that may provide clues to function such as phylogenetic profiles, expression profiles, and profiles of CRISPR phenotypes as in DepMap. Thus, we feel that it would be best to leave this to future studies and indeed are currently applying machine learning to combine such data sources to make predictions about function. In the meantime, the Unknome database provides a link to the UniProt entry for every protein in every cluster, and these entries have links to protein-protein interaction databases. This was not clear as the relevant column on the Cluster page was labelled “Protein ID” and so we have changed this to “UniProt ID”.

Reviewer #3:

This manuscript by Rocha, et al. uses extensive bioinformatics and statistical analysis to assign scores for orthologous protein clusters found in humans and at least one other model organism to raise awareness of uncharacterized and understudied proteins. The authors created a publicly available database where users can assess a so-called 'knownness score' for proteins of interest based upon multiple factors, including GO terms or experimental knowledge. A group of proteins with unknown gene function (>350) were subjected to RNAi analysis in Drosophila to assess possible function related to seven categories - viability, fertility, wing growth, response to stress, locomotion, and protein aggregation. The database was a Herculean amount of work and provides a valuable resource for the scientific community, especially if the website will be updated and maintained. Improvements in the manuscript are required, especially toning down the language that overstates some of the conclusions. The value of this paper is in the database, not necessarily the biological experiments.

(1) What is the timeframe for this study and/or development of the website? The phrase 'during the course of our studies' is used and it is unclear how long these efforts took.

The database was developed at the start of the project, and the website established about three years ago, but the database and website have been kept up to date with new releases of Panther, GO, UniProt etc. The experimental work took several years as would be expected for large scale phenotypic screens. The validating and write up took more time than hoped due to the pandemic and some of its consequences, but these are perhaps not things to be discussed in a scientific paper. Thus, to capture this, we have referred to the "protracted" course of our studies when this phrase is first used.

(2) Social aspects is described as a factor that may promote scientific bias. The meaning of this is not clear and likely not a valid argument without supporting data. Please remove.

We have removed the word "social" from the abstract.

(3) Figure 1F is not referred to in the manuscript.

We apologise for this error – it was referred to as Figure 1G, and this has now been corrected.

(4) Many of the graphs would benefit from descriptive titles. For example, Figs 2A and S2A look similar, yet compare human vs non-human model organisms separately. Also true for tables such as 1E and 2C.

We agree that this would add clarity to the figures and titles have been added to all the graphs and tables in Figures 1, 2, S1 and S2.

(5) da-Gal4 is a weak ubiquitous driver and should be noted as such. Likely more RNAi lines would be lethal if a stronger driver was used.

We initially tested very strong drivers such as actin and tubulin and found that they could cause lethality with RNAi hairpins from known non-essential genes. In contrast, the more moderate da-Gal4 gave more accurate results with known essential and non-essential genes, and it has been used successfully for other RNAi experiments in whole flies. We agree that the choice of driver is important, and so we have now noted in the Results that we used da-Gal4, so that readers are fully aware of our approach.

(6) Please include the rationale in the text for the controls utilized for fertility analysis and locomotion. Also include controls for Figures 4B and 5F. They provide a nice comparison for the effects seen.

We have added a note to the figure legends to explain the use of these control proteins along with relevant citations. We initially incorporated controls to help set up some assays we had not used before, and these are shown for information. However, we did not incorporate such controls in all the assays as we did not rely on control stocks for statistical determination of outliers, but rather relied on the much larger amount of information provided by the variation amongst all of the lines that were screened as this covers many more lines and so gives a more accurate measure of the degree of variation that is intrinsic in the assay and hence allows a more reliable means to identify outliers. This is a commonly used approach in large-scale RNAi screens, and this is briefly outlined in the Results with the details of the statistical analysis summarised in the Materials and Methods and provided in full in the statistical supplement.

(7) The axes for Figure 3D should be in a distance measurements, not pixels.

The quantitation of the area of intervein regions of *Drosophila* wings was based on the pixels that comprise the images and so these are the units used in the data. To make clear what distance this corresponds to, we now state in the figure legends that the pixel dimensions are 2.5 µm x 2.5 µm.

(8) A better resolution picture is needed for Figure 4A as the current one looks grainy. Representative pictures for the positive hits in panel 4B should also be included.

We apologise for this poor resolution image and have replaced it with a full resolution image along with a representative image of a hit from the screen.

(9) Is there confirmation of reduced mRNA and/or protein levels for any of the CRISPR/Cas9 mutants?

There were no suitable antibodies available to detect the proteins knocked out by CRISPR/Cas9 and one might not expect mRNA levels to always be affected. All the CRISPR/Cas9 indels that gave a phenotype were tested over deficiencies of the corresponding region and so it seems certain that there is a reduction or loss of the expressed protein. It is formally possible, if perhaps unlikely, that the indels that did not show a phenotype left sufficient residual or truncated protein that prevented the appearance of even a partial phenotype. We have added a caveat to this effect to the section on CRISPR/Cas9 in the Methods.

(10) The following statement 'Male flies lacking CG10064 produced motile sperm, but following mating they did appear to not persist in the female's sperm storage organ, the seminal receptacle' is not supported by any data.

This is a minor point and so for the sake of brevity we have removed this statement.

(11) Is there published literature to support the statement 'suggesting a true hit rate of ~50%, a reasonable outcome for an RNAi-based approach.' Is it true that 50% of RNAi screens are hitting off-target? This seem unlikely and should be modified.

We agree that it is difficult to be confident about what is a reasonable outcome from an RNAi screen, and also the number of genes used to make this estimate is small, and so we have removed this statement.

(12) The word 'ignorance' is used multiple times throughout the manuscript and is misleading. While the definition of ignorance is a lack of knowledge, the word implies uninformed or lack of education. As scientists, all of us want knowledge. Just because something has not been studied previously does not mean the community is ignorant. Please change this language throughout the manuscript, especially in the title.

We agree that 'ignorance' is sometimes abused in reference to individuals. However, in a scientific context it is generally applied to the sum of human knowledge, and there is a long tradition of it being used in this manner by other scientists. For instance, there is a book by Stuart Firestein called "Ignorance: How it Drives Science" (Oxford University Press, 2012), based on a lecture course he gives at Columbia University. We are confident that neither university would tolerate language that was offensive. In addition, the word 'ignorance' has appeared in the title of 923 papers in PubMed dating back to 1854. Finally, the word has been used in the context intended here by several of the most eminent scientists including Schrodinger "In an honest search for knowledge you have quite often to abide by ignorance for an indefinite period" and Maxwell "Thoroughly conscious ignorance is the prelude to every real advance in science". Thus, we feel that our use of the word 'ignorance' is entirely appropriate. To make it absolutely clear to readers that we do not intend offence we have

included a reference to Firestein’s book and the quote from Maxwell as they underline the potential value of what we have tried to do.

(13) The abstract (and elsewhere) states the present work demonstrates the importance of poorly understood genes. This isn't new knowledge or surprising data. It is understood that many uncharacterized genes likely have important functions. Please change this type of language throughout the manuscript. The focus should be on the website.

Strictly speaking, the word ‘demonstrate’ does not mean to make a new or surprising finding, but rather it means to illustrate or exemplify some aspect of knowledge. Moreover, whilst it may be understood by some that many uncharacterised genes are likely to have important functions this has not prevented most current research focusing on well characterised genes, nor has it prevented an apparent bias in funding and even career progress such that both favour work on well characterised genes (as shown in studies we cite in the manuscript). Thus, we feel that the point is worth highlighting. However, to avoid ambiguity, we have altered two of the three occurrences of “demonstrate” in the paper. In the abstract it is now “illustrates”, and in the discussion is it is now “provides further evidence”.
