# Supplementary material for: Functional unknomics: Systematic screening of conserved genes of unknown function
Source: PLoS Biol. 2023 Aug 8;21(8):e3002222. doi: 10.1371/journal.pbio.3002222 (PMC10409296; doi:10.1371/journal.pbio.3002222)
Supplement: Attachment — Submitted filename: Response to Reviewers D-23-00105R2.docx [file pbio.3002222.s013.docx]

**Response to Editorial Requests.**

(A) We would like to suggest the following modification to the title:

“Functional unknomics: in vivo screening of conserved genes of unknown function reveals the importance of poorly understood genes”

The title in the revised manuscript has been changed in line with this suggestion (although I think that it would convey much the same thing more succinctly if the words “of conserved genes of unknown function” were removed.

However, we would like to make one final plea for using our original title, especially as it is not clear to us why you feel it should be changed. Is it because it includes the word “ignorance”? If so, then we thought that we had provided a reasonable defence of this in our reply to Reviewer 3.

We would really like to use our original title for what is, after all, our work. The main reason is that we strongly feel that it would help make the paper more visible. We would note that a short essay in the Journal of Cell Science on ignorance in science had an even more blunt title: <https://doi.org/10.1242/jcs.033340> This essay has been viewed over a million times, which is surely evidence of the benefits of an eye-catching title!

(B) You may be aware of the PLOS Data Policy, which requires that all data be made available without restriction: http://journals.plos.org/plosbiology/s/data-availability. For more information, please also see this editorial: http://dx.doi.org/10.1371/journal.pbio.1001797

Note that we do not require all raw data. Rather, we ask that all individual quantitative observations that underlie the data summarized in the figures and results of your paper be made available in one of the following forms:

-Supplementary files (e.g., excel). Please ensure that all data files are uploaded as 'Supporting Information' and are invariably referred to (in the manuscript, figure legends, and the Description field when uploading your files) using the following format verbatim: S1 Data, S2 Data, etc. Multiple panels of a single or even several figures can be included as multiple sheets in one excel file that is saved using exactly the following convention: S1_Data.xlsx (using an underscore).

-Deposition in a publicly available repository. Please also provide the accession code or a reviewer link so that we may view your data before publication.

The individual quantitative observations that underlie are the data in the paper are provided in the Supplementary Files. The entire Unknome database can be downloaded from the database website.

Regardless of the method selected, please ensure that you provide the individual numerical values that underlie the summary data displayed in the following figure panels as they are essential for readers to assess your analysis and to reproduce it:

Figure 1F, 2A-B, 3A, 3D, 4B, 4E-F, 5C, 6C-D, S1B, S2A-E, S3B, S3D, S4A-B

The individual numerical values for all of the indicated Figure panels are provided in supplementary file S1 Data, with the exception of 3A, 3D, 4B, 4F and 5C which are the plots of the genetics screens, with this data is provided in S2 Data.

(C) I appreciate that much of the underlying data that I have requested above is already provided in the S1 Data file. I would be grateful if this file could be annotated so it is made clear which specific figure panels relate to each dataset in the file.

We have annotated the two numerical value files (S1 Data and S2 Data), so that the relevant figure panel is stated in the tab of each sheet in the Excel file.

(D) In addition, I note that a S2 data file is referred to in the manuscript, but there does not appear to be a S2 data file in the File Inventory. I would be grateful if this could be provided upon resubmission.

We apologise for this error, and the file is now back in the File Inventory (now S3 Data). It contains the raw data that underlies all of the genetic screens.

(E) Please also ensure that each of the relevant figure legends in your manuscript include information on where the underlying data can be found, and ensure your supplemental data file/s has a legend.

We have added a note to each figure legend to indicate where the underlying data can be found. We have provided legends for all of the supplemental data files – these are at the end of the manuscript file under the heading “Supporting Information”.

(F) Please ensure that your Data Statement in the submission system accurately describes where your data can be found and is in final format, as it will be published as written there.

We have checked the Data Statement to ensure that it accurately describe where the data can be found.
